# Sleep and diurnal alternative polyadenylation sites associated with human APA-linked brain disorders

**DOI:** 10.21203/rs.3.rs-4707772/v1

**Published:** 2024-08-08

**Authors:** Carlos C. Flores, Nickolas A. Pasetto, Hongyang Wang, Alexander G. Dimitrov, Jon F. Davis, Zhihua Jiang, Christopher J. Davis, Jason R. Gerstner

**Affiliations:** Washington State University; Washington State University; Washington State University; Washington State University; Novo Nordisk (United States); Washington State University; Washington State University; Washington State University

## Abstract

Disruption of sleep and circadian rhythms are a comorbid feature of many pathologies, and can negatively influence many health conditions, including neurodegenerative disease, metabolic illness, cancer, and various neurological disorders. Genetic association studies linking sleep and circadian disturbances with disease susceptibility have mainly focused on changes in gene expression due to mutations, such as single-nucleotide polymorphisms. The interaction between sleep and/or circadian rhythms with the use of Alternative Polyadenylation (APA) has been largely undescribed, particularly in the context of other disorders. APA is a process that generates various transcript isoforms of the same gene affecting its mRNA translation, stability, localization, and subsequent function. Here we identified unique APAs expressed in rat brain over time-of-day, immediately following sleep deprivation, and the subsequent recovery period. From these data, we performed a secondary analysis of these sleep- or time-of-day associated PASs with recently described APA-linked human brain disorder susceptibility genes.

## Introduction

Dysregulation of sleep and circadian rhythms can profoundly impact human health and compound disease^1,2^. Indeed, sleep disruption is associated with negative outcomes in cardiovascular, metabolic, immunologic, and cognitive health that can have substantial short- and long-term consequences^3^. Alterations in sleep and circadian rhythms are often observed with various brain disorders, including autism spectrum disorder, bipolar disorder, major depression, schizophrenia, Parkinson’s, and Alzheimer’s diseases^4–7^. Complicating the association between sleep and health is the fact that functional aspects of sleep remain largely undefined and inconclusive^8,9^; however, the use of evolutionarily distinct animal models to study sleep has historically offered keen insights^10,11^. For example, studies on circadian- and sleep-dependent gene-regulatory mechanisms in diverse species, including flies, rodents, and humans, have identified important phylogenetically conserved pathways with functional relevance^12–15^. Employing unbiased approaches, such as large-scale metabolomic, transcriptomic, and proteomic analyses, have also greatly aided in the generation of conceptual frameworks for characterizing sleep function in health^14,16^. Therefore, performing such discovery-based studies of sleep and circadian regulatory processes in model organisms will help define the fundamental biological mechanisms underlying sleep function and inform pre-clinical relevance for comorbidities of sleep dysfunction associated with poor health.

Alternative polyadenylation (APA) site usage is an important and often overlooked mechanism of gene regulation, that can affect mRNA stability, mRNA/protein targeting, translational competence, and generate alternative protein isoforms^17,18^. APA sites are common and occur most frequently in the 3’ untranslated region (3’ UTR) of mRNAs across phylogeny, with more than half of human genes having multiple polyadenylation sites (PASs) that generate alternative isoforms^19^. These isoforms can have altered coding sequences or 3’UTRs, resulting in the diversification of cis-regulatory elements (e.g., RNA binding protein sites, microRNA binding sites) that influence transcript abundance, trafficking, stability, and/or translation efficiency^20^. Furthermore, there’s growing evidence of cell-type-specific APA preference^21^. The involvement of APA in the context of sleep and circadian rhythms has been largely unexplored, with the few studies available mostly focused on peripheral organs^22,23^ and cells^24^. Here, we have characterized how APA site usage oscillates based on the time of day as well as how it is altered following acute changes in sleep pressure, specifically in the adult mammalian brain. Multiple methodologies have been developed for transcriptome-wide profiling and mapping of APA sites^25,26^. To complete this study, we performed whole transcriptome termini sequencing (WTTS-seq)^27,28^ analysis to profile the variations in APA usage that occur due to sleep pressure and daily rhythms in the rat forebrain. Over 31,000 PASs were recovered in total, with 45% of the represented genes having multiple APA sites. Interestingly, many of the PASs sequenced were not previously annotated in the rat genome. Moreover, a total of 2,011, (6%) of PASs cycled over the day, and 831 (3%) were homeostatically regulated following sleep loss following sleep loss or during recovery. Over half of all cycling or differentially expressed PASs were APAs, (i.e., in genes with ≥ 2 PASs). Given the importance of sleep^4–7^ and APA in health and disease^25,29,30^, we compared our sequencing results with results from a recent study that determined APA usage in human brain disorder susceptibility^31^. The genes found in both studies warrant further examination and could lead to new preclinical animal models to investigate these disorders.

To the best of our knowledge, the current study represents the first comprehensive, transcriptome-wide mapping of APA sites in adult mammalian brain tissue over the day-night cycle as well as following changes in sleep homeostasis. This global temporal dataset will be useful for future comparative studies that require the determination of baseline APA site usage profiles in the mammalian brain. Furthermore, our study underscores the importance of using alternative-omic approaches to characterize phylogenetically conserved genome-phenome information and reveals another expansive layer of complexity in sleep and circadian gene regulation that has not previously been documented.

## Results

### Identification of PASs in the rat forebrain.

**Figure 1:**
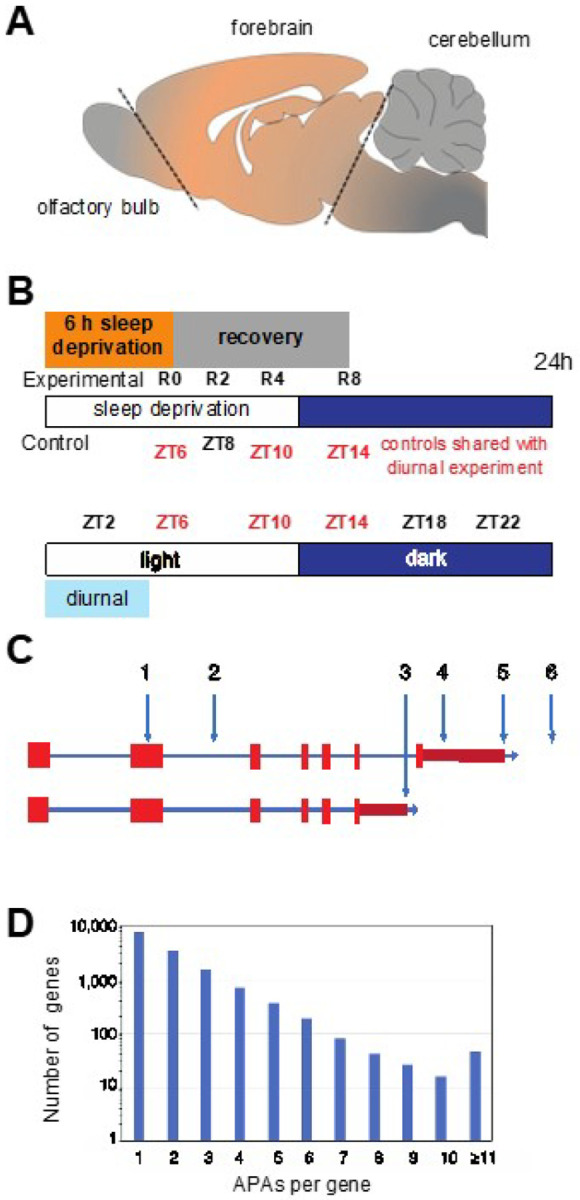
Schema of the brain region sampled, the collection time/condition, and a plot of the number of APA sites per gene. **(a)** The region of the central rat forebrain that was collected and used for RNA extraction is bounded by dotted lines and labeled ‘forebrain’. **(b)**For sleep homeostasis experiments, rats were sleep-deprived for 6 h and allowed to recover for 0 to 8 h before tissue extraction. Three of the time-matched controls (no SD) were shared with the diurnal experiment and one additional time point (no SD at ZT8), was not in common. For the diurnal analysis, samples were taken at 4 h intervals from ZT2 until ZT22. Five biological replicates were used for all data points. **(c)** A diagram of a generic gene shows different types of APAs: within an internal exon (1); within an early intron (2); following an internal exon (3); within the longest documented 3’ UTR (4); at the terminus of the longest documented 3’ UTR (5); and distal to longest documented 3’ UTR. **(d)** WTTS-seq PAS results; the number of genes on the x-axis (log10 scale) are plotted against the number of APA sites per gene.

Given the rat transcriptome is not as extensively annotated as the human or mouse, we first identified all PASs, including novel candidate PASs prior to determining changes in PAS usage. Replicate diurnal (central forebrains) were taken from five rats every four hours starting at two hours after lights on (i.e., ZT2, ZT6, ZT10, ZT14, ZT18 and ZT22) (Fig. 1a, b). RNA was purified from these samples and used to generate WTTS-seq cDNA libraries that were subsequently sequenced. Poly(A)-directed sequence reads were then mapped to the rat genome, giving rise to 31,757 PAS clusters (see Supplementary Table S1). Among the 31,757 PAS clusters identified, a sizable portion mapped to novel unannotated PASs, leaving 26,635 PASs that mapped to named loci (i.e., genes). Many APAs occur at different points within the longest 3’ UTR (Fig. 1c, sites 4 and 5). Some are distal to the longest documented 3’ UTR (site 6), while some occur in internal exons (site 1) or introns (sites 2 and 3) (Fig. 1c). In our data set of all PASs that mapped to genes, 45% mapped to genes with ≥ 2 APA sites, and 19% mapped to genes with ≥ 3 APA sites (Fig. 1d).

### Identification of PASs that exhibit a daily cycle.

Periodicity of PAS expression was assessed using meta2d^32^. Diurnal (24 h period) oscillations were demonstrated for 2,011 PASs. Among these, 1,173 were in genes with ≥ 2 total APA sites, including ones in known diurnal transcripts, such as *Dbp* (diurnal in 2 of 2 APA sites recovered), *Nr1d2* (in 1 of 1), *Per2* (in 2 of 2), and *Ntrk2* (in 2 of 10)^33^ (Table 1 and Supplementary Table S2).

We were interested whether rhythmic PASs might cluster predominantly into certain phases of peak expression, and whether APAs that share a common peak phase might also share some functional relationship. It was evident that some phases had very few APAs relative to other phases and the expression levels of many PASs peaked around ZT18–20 (Supplementary Fig. S1). When diurnal APAs from genes with ≥ 2 total APAs were grouped by phase, GO and pathway analysis on each group found that only phases 2, 10 and 18 had significantly over-represented terms. Phase 18 had the most, with the over-representation of multiple signaling pathways, including ‘neuron to neuron synapse’ and ‘post-synaptic specialization’ (Supplementary Table S4).

There is a growing appreciation that rhythms shorter than 24h are biologically relevant^35–39^. Thus, we evaluated the PASs data for ultradian cycling using meta2d with the period set to 12 h. Overall, 1,502 PASs that cycled with a 12 h period were identified (Supplementary Table S5). Of the 12 h cycling PASs, 1,198 were in genes, and after adjusting for genes with multiple 12h cycling APAs, there were 1,149 unique genes in the set. In total, 827 of the 12 h cycling APA sites were in genes that had ≥ 2 APAs, representing 778 unique genes. Pathway analysis on this set of 778 unique genes (Supplementary Table S6) showed that CREB phosphorylation and circadian entrainment were highly enriched, while GO analysis of this data set resulted in 16 GO terms related to the synapse.

### PASs are differentially expressed after sleep deprivation and during recovery sleep.

**Figure 2:**
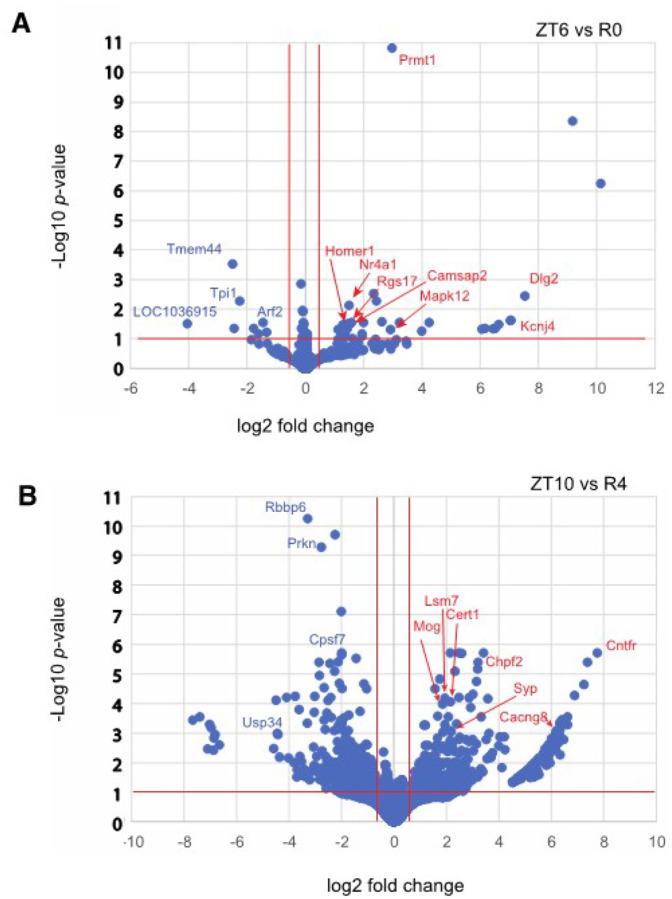
Differential expression of PASs by DESeq-2 with Apeglm Shrinkage. Log of adjusted *p*-values are plotted against log2 fold changes from **(a)**ZT6 vs R0 and **(b)** ZT10 vs R4.

To investigate changes in APA site usage related to sleep pressure, rats were subjected to SD for 6 h from ZT0 to ZT6, and central forebrain tissue was collected immediately afterwards (R0). Additional animals were allowed to recover for 2, 4, or 8 h after SD (R2, R4 and R8) before tissue was collected. WTTS-seq data from these samples were compared to time-matched controls that were allowed to sleep undisturbed (ZT6, ZT8, ZT10 and ZT14). All groups consisted of 5 biological replicates. Our sequencing data showed that the most significant differences in expression were seen when we compared R0 with its control (ZT6) and R4 with its control (ZT10) (Supplementary Table S7 and Fig. 2). Interestingly, a *Homer1a* APA isoform is the most abundant at R0, R4 and ZT6, whereas a full-length isoform is dominant at ZT10 (Supplementary Fig. S2 a and b) Also, the expression of one APA isoform of *Prmt1*, was upregulated with high confidence after 6h of sleep deprivation (Fig. 2). PRMT1 protein regulates multiple stress response pathways^40,41^, which have a roll in acute sleep loss.

The gene names of differentially expressed APA sites from genes with ≥ 2 APAs were used for GO and pathway over-representation analysis (Table 3). ZT6 vs R0 only had significant results for GO while ZT10 vs R4 had significant GO and pathway results.

## Discussion

APA site usage is an understudied aspect of gene regulation. Although APA sequencing can reveal changes in overall gene expression, it’s designed to focus on changes in APA usage and cannot reveal differences in splicing or transcription start sites (TSSs). On the other hand, bulk RNA-seq analysis often ignores APA, TSS and splice isoforms to simply assess reads per gene. Currently it would be very difficult to enumerate copies of all the mRNA isoforms for each gene. Yet appreciation is growing for the importance of APA sites in regulating mRNA stability^17,42^, mRNA/protein localization^20,43,44^, and human disease^31,45^.

Rhythmic APA site usage has been uncovered in the mouse liver^22,23,46^, and in temperature-entrained cultured cells, circadian APA usage occurs in many genes and can regulate expression of specific central clock genes^24^. Still, alternative poly(A) site usage hasn’t been given much attention in the sleep and circadian field. We therefore initiated this investigation into the conjunction of APA with sleep and diurnal expression. As far as we are aware, the current study is the first to examine APA sites related to circadian rhythms and sleep pressure in any mammalian brain. There are several, diverse ways in which data from this study can translate into biological relevance as described in the examples below.

**Figure 3:**
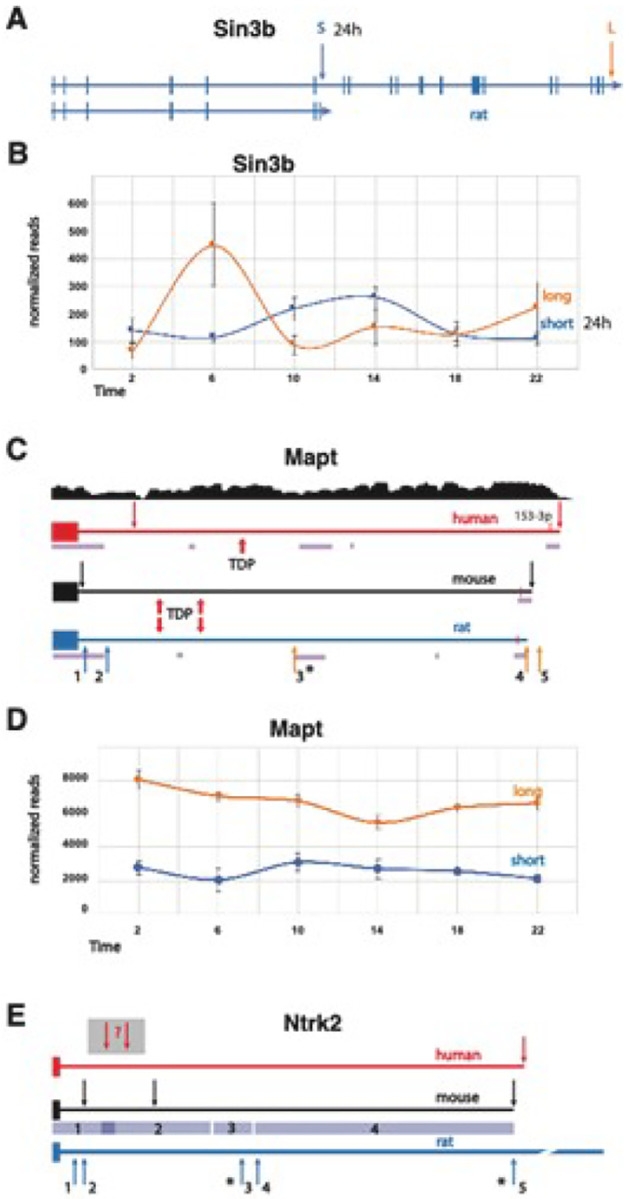
The *Sin3b* gene and 3’ UTR regions of the *Mapt* and *Ntrk2* genes. **(a)** A map of the entire rat *Sin3b* gene depicts exons, introns and short and long APA sites. The corresponding genes in mouse and human are extremely similar. **(b)** The average normalized read counts ±SE (y-axis) of the short (diurnal) and long *Sin3b* APAs are plotted against time-of-day (x-axis). **(c)** Maps of the 3’ UTR regions of the human, mouse, and rat *Mapt* genes are shown. Arrows labeled 1–5 indicate the positions of APA sites. In human *MAPT*, APA usage correlates with several brain disorders. RNA-seq coverage from individuals homozygous for the less common SNP allele that is associated with longer transcripts (adapted from Cui. et al.^27^) is shown above the human *MAPT3’* UTR map. Binding sites for TDP-43 (indicated by red arrows) that were experimentally determined in mouse align with putative sites in the rat gene, and one possible TDP-43 binding site is indicated in the human 3’UTR. The significantly diurnal APA is marked with an asterisk. Blocks of homologous sequence between the rat and human genes that were found by BLAST search are indicated by purple bars. The 3’ UTR lengths are 4,380, 4,119 and 3,946 n.t. for human, mouse, and rat, respectively. **(d)** The average normalized read counts ±SE (y-axis) of the short *Mapt* isoforms lacking TDP binding sites (1+2) and the sum of the three longer isoforms (3+4+5) plotted against time-of-day (x-axis) are shown. **(e)** The 3’ UTR of tyrosine kinase-deficient (TK−) isoforms of the human, mouse, and rat *Ntrk2* TK− genes are shown. Arrows indicate the positions of APA sites. The depicted rat APAs are from this current dataset. Diurnal rat APAs are indicated with asterisks. The 3’ UTR lengths are 5,125, 5,008 and 8,004 n.t. for human, mouse, and rat, respectively. Mouse and rat sequence comparison by BLAST produced 4 segments having 91%, 83%, 86% and 82% identity for regions 1, 2, 3 and 4, depicted by blue bars.

Here, we observed that 6% of all PASs cycled with a 24 h period. One of the top pathways identified for the diurnal APA gene set was ‘circadian entrainment’ (Table 2). Since transcription-translation feedback loops are central to circadian regulation, this may not be surprising, but APA site usage suggests a more complex role^24,46^. For example, we find that one *Sin3b* APA follows a diurnal rhythm (Fig. 3a, b). *Sin3b* encodes short and long variants conserved in mammals. The short variant binds to CRY1 but cannot bind HDAC1^47^. The long isoform is implicated in regulation of Per1/Per2 transcription^48^, along with many other genes^49^. In our data, long *Sin3b* APA reads constitute the predominant isoform at ZT6 and ZT22, while the short, diurnal isoform is the most abundant one at ZT10, ZT14 and perhaps ZT2 (Fig. 3b). *Sin3b* transcript levels in mouse hippocampus have previously been reported to be affected by sleep deprivation^50^, although this effect was not observed using TRAP-seq^51^, suggesting post-transcriptional processing can lead to changes in sleep-dependent differential expression. Together with our work, this example highlights the importance of utilizing various “-omic” approaches to properly decipher the complexity of molecular processing tied to changes in behavioral state in the brain.

Additional significant pathways emerged from the diurnal APAs, such as Oxytocin, Ephrin, and MAPK signaling that have demonstrated links to the circadian clock^52–54^. In the GO analysis of the diurnal genes with multiple PASs, we discovered that terms related to the synapse (12), protein localization (6), and vesicles (7) (Table 2 and Supplementary Table S3) were enriched suggesting APAs are poised to affect neural communication.

A large proportion of diurnal APAs had expression peaks around ZT20 (Supplementary Fig. S1). Considering that rats are nocturnal, this is similar to what has been seen for bulk transcripts in several human tissues, including brain^55^. Interestingly, among the identified diurnal APA sites, 3 were in genes for RNA-binding proteins (*Celf2*, *Elavl3*, and *Rbfox1*) whose expressions correlate with more distal APA usage^47^. Peak expression of these three genes is from ZT21 to ZT1, so it would be interesting to see if transcripts of predicted targets tend to be longer at these times.

In addition to the 24 h circadian rhythm, recent studies have also demonstrated the existence of cell-autonomous ultradian clocks that run independently of the circadian clock to regulate 12 h oscillations in gene expression and metabolism^35–39^. Here we found that 5% of all PASs cycle with a 12 h period. Further analysis of these genes showed enrichment of gene ontology terms and pathways such as “regulation of trans-synaptic signaling” and “protein-protein interactions at synapses” (Supplementary Table S6), indicating that APAs could function to regulate cyclic actions of cell signaling and communication.

Gene expression studies following changes in sleep homeostasis have largely ignored alternative polyadenylation. Of the 31,795 total PASs characterized in rat forebrain in our study, we determined that 2.5% were differentially expressed with sleep deprivation and recovery sleep. We also observed 6 GO terms significantly enriched following 6 hours of sleep loss and 26 following 4 hours of recovery sleep (Table 3).

Human APA isoforms have been linked to many neurological disorders^31^. Among the genes that we identified to have rhythmic expression of APA sites or had APA sites that were affected by sleep pressure, we found that 46 have also been correlated with brain disorder susceptibility (Table 4). For example, the human *MAPT/TAU* gene produces transcripts containing short or long 3’ UTRs, and a 3’ single-nucleotide polymorphism, (SNP) is associated with both 3’ UTR length and risks for 8 neurological disorders, including Alzheimer’s and Parkinson’s diseases^31^. Homozygosity of the more common SNP variant is associated with short *MAPT3’* UTRs, homozygosity of the less common SNP variant is associated with long 3’ UTRs, and heterozygosity is associated with 3’ UTRs of intermediate lengths. In our rat APA data, there were both short and long 3’ UTR forms (5 in total) of the *Mapt* gene that were identified (Fig. 3c, d). Only two are currently annotated in the rat genome and one of the newly discovered APAs was observed to cycle with time-of-day. In mouse, binding of the ALS-associated protein TDP-43 to two sites in the 3’ UTR of *Mapt* has been shown to destabilize the mRNA^56^. In Alzheimer’s disease, the expression level of TDP-43 protein is often low, and TAU is overexpressed and eventually forms neurofibrillary tangles. The two TDP-43 binding sites that were experimentally determined in mouse are conserved in sequence and position in the rat gene, implying that transcripts with shorter 3’ UTRs would not be affected by TDP-43, while longer ones could be destabilized^56,57^. The presence of at least one putative TDP-43 binding site in the human MAPT 3’UTR suggests that this may be contributing to the neurological disorder risk.

*Ntrk2* is among the APA TWAS genes linked to anxiety^31^ and has been associated with autism in other studies^58^. We found strong time-of-day oscillations of the 2 most abundant APA sites of the short, tyrosine kinase deficient (TK−) *Ntrk2* isoform. The TK− isoform of *Ntrk2* has several known functions, including a dominant negative effect on the full-length TK + isoform during neuronal proliferation, differentiation, and survival. In addition, the TK− version promotes filopodia and neurite outgrowth; sequesters, translocates, and presents BNDF; and affects calcium signaling and cytoskeletal modifications in glia^59^. Our WTTS-seq data revealed short, medium, and long 3’ UTRs in the rat *Ntrk2* TK− isoform (Fig. 3e). In mice, the longer *Ntrk2* TK− transcripts are preferentially targeted to apical dendrites^60^. Since the sequence of the rat 3’ UTR is highly conserved with the mouse sequence, it is plausible that an analogous dendritic localization mechanism is also in use in the rat (Fig. 3e). Interestingly, ‘Ntrk signaling’ was one of the pathways over-represented in the diurnal APA genes (Supplementary Table S3). APA sites in *Src, Frs2, Atf1, Nras, Sh3gl2, Ntrk3, Mapk1, Grb2, Pik3r1*, and *Mapk14* contributed to this enrichment.

**Figure 4:**
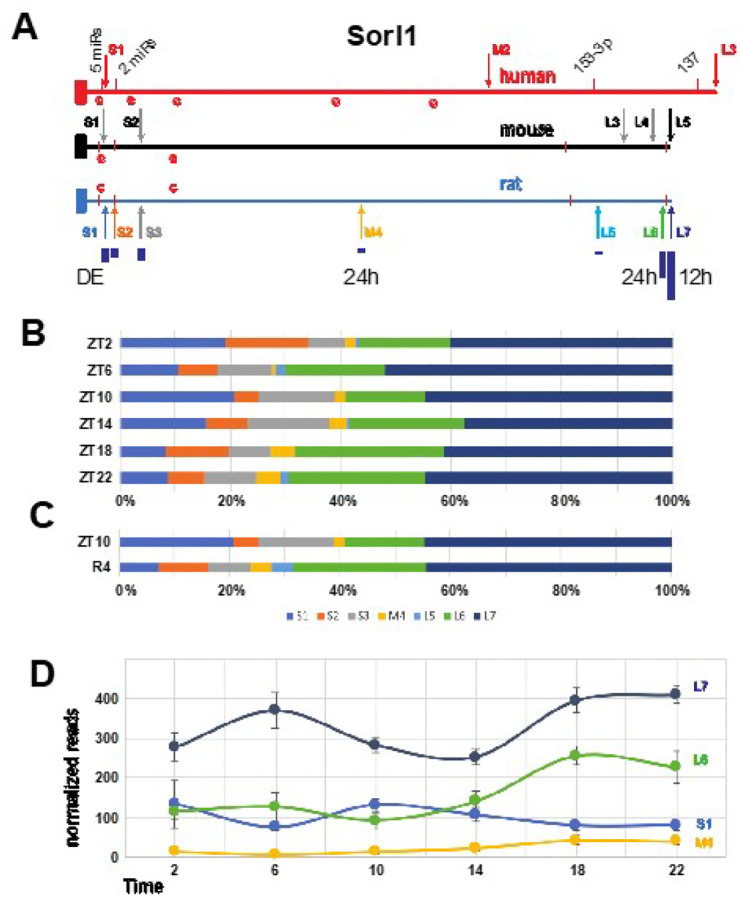
Map and APA read analyses of *Sorl1*. **(a)**Maps of the human, mouse and rat *Sorl1* gene 3’ UTRs show APA sites indicated by arrows. Four highly conserved miR binding sites are marked by red bars in all three species. The first 2 are recognized by multiple miRs. The size of dark blue bars under the rat APAs depict the individual proportion compared to the total of all WTTS *Sorl1* reads. The human APAs are from established isoforms which also include different exon configurations. The first 4 mouse APAs are suggested by ESTs, and, in the latter 3 cases, by upstream polyA signals and PolyA_DB v3 data. Red ‘c’s indicate matches to the consensus CPE sites. **(b)** The proportion each *Sorl1* APA contributes to the total for the gene are plotted for each of the diurnal timepoints. **(c)** The proportion each *Sorl1* APA contributes to the total for the gene are plotted for the differentially expressed samples: ZT10 and 4 hours after SD. **(d)** Graph of normalized read numbers of 4 *Sorl1* APAs that either cycle with 24 h (M4 and L6) or 12 h (L7) hours and the one differentially expressed after SD (S1).

Four different APAs from the *Sorl1* gene exhibited significant changes in our analyses; two diurnal, one cycled with a 12 h period, and one was reduced during recovery from sleep deprivation (Fig. 4). In total, there were seven APAs in the Sorl1 3’UTR, three short, one medium and two long. The longest and most abundant isoform cycles per 12 h, the second longest and medium ones are diurnal and the shortest isoform is differentially expressed after SD (Fig. 4). SORL1 encodes an endosomal recycling receptor^61^, and a deficiency of *SORL1* as well as many polymorphisms are strong risk factors for AD^62,63^. The mouse and human 3’ UTRs share extensive similarities including 5 APAs in mouse and 3 in human based on the PolyA_DB v3 (https://exon.apps.wistar.org/polya_db/v2/) and UCSC database^64^. Four microRNA binding sites with high probability of preferential conservation are in good alignment (TargetScanHuman v8.0)^65^. The first motif can be bound by five miRNAs (miR-25–3p, miR-32–5p, miR-92–3p, miR-363–3p, and miR-367–3p), while the second contains overlapping 7mer and 8mer motifs bound by miR-128–3p and miR-27–3p, respectively. The final two more distal sites are recognized by miR-153–3p and mir-137 (Fig. 4a). Sequences matching the consensus binding site for CPEB are present in the 3’ UTRs of all three species, with 2 in very good alignment. Cytoplasmic polyadenylation element binding protein (CPEB) facilitates mRNA trafficking to synapses and local translation^66,67^, and we have previously shown that the core clock-controlled *Fabp7* mRNA^68,69^ contains functional CPE sites in its 3’UTR to regulate translation^70^. Since APOE4, an apolipoprotein E variant with increased risk of AD^71^, disrupts FABP7 interaction with sortilin, (an APOE receptor similar to Sorl1), to interfere with neuroprotective lipid signaling^72^, this suggests circadian variation in local translation of CPEB-mediated polyadenylation of target mRNAs may be a generalizable mechanism that modulates AD susceptibility through downstream lipid pathways. Any one or more of these conserved features could lead to conserved functional consequences dependent on APA choice.

One caveat to our approach is that WTTS-seq generates Ion Torrent PGM sequences which may retain more noise compared to Illumina platform reads and since only Illumina has the option of paired-end reads, there can be more uncertainty in mapping Ion Torrent reads. Our strategy was to capture the maximum number of PASs, including the discovery of novel PASs, and the rat genome is not as thoroughly annotated as some other vertebrate species, we therefore included potentially intergenic reads. In our analysis, we found 5,122 PASs and 318 diurnal PASs that mapped outside of known genes, and many APAs within genes mapped to regions in which 3’ ends have yet to be annotated. Based on prior WTTS-seq data sets and other PAS mapping approaches, some portion of our PASs could be method-based artifacts^27,73^, (see Zhou et al.^27^ Figs. 3, 4 and 5). In this, our initial PAS survey, we assayed a large portion of the brain. Therefore, future studies in restricted brain structures or cell types will be required to uncover APAs that cycle or are differentially expressed at a finer scale. Overall, the newly discovered PASs should add valuable insights into regulation of the rat transcriptome and for characterizing PAS usage in the mammalian brain.

Here we used an unbiased discovery-based approach for uncovering novel APA usage following time-of-day or changes in sleep pressure in mammalian brain. These data leverage a call to action for additional work to elucidate the core mechanisms of PAS usage in the brain and to examine the capacity of APA to affect the transcriptomes and proteomes that regulate central brain processes known to be altered by time-of-day and sleep/wake homeostasis. Moreover, it known that PAS usage varies across brain region and cell type^21^ (i.e., substructure-, circuit-, laminar- or nucleus-specific)^74^. These hypothesis-generating data provide an impetus for continued research aimed at delineating how sleep and circadian rhythms impact mental health and neurodegenerative disease.

## Methods

### Subjects.

All animal procedures were carried out in accordance with the National Institutes of Health Guide for the Care and Use of Laboratory Animals and ARRIVE and OLAW guidelines and approved by the WSU Institutional Animal Care and Use Committee (IACUC; ASAF# 6804). Male Long Evans rats (7–9 weeks old) were housed in pairs at 22 ± 2°C on a 12:12 h light-dark cycle. The rats were acclimated to this light cycle for at least 10 days prior to tissue collection, with water and chow *ad libitum*. Cages were cleaned weekly (between 8 and 11 AM) unless the rats were being euthanized within 24 h. Thirty rats were randomly assigned to one of six groups (*n* = 5/group) that were sampled every 4 h, beginning 2 h after light onset (zeitgeber time (ZT)) (i.e., ZT2, 6, 10, 14, 18, and ZT22). For the sleep deprivation (SD) study, twenty rats were randomly assigned to 6 h SD from ZT0–6, wherein rats were kept awake by an automated bedding stir bar (Pinnacle) at the bottom of a cylindrical cage. The bar was set to rotate for 4 s, randomly changing rotation direction, and stopped for a random interval ranging from 10 to 30 s^75,76^. Following SD, rats (*n* = 5/time point) were euthanized immediately (R0) by live decapitation or were returned to their home cage for 2 h (R2), 4 h (R4), or 8 h (R8) under red light without disruption before sampling. Five additional rats were euthanized at ZT8 as undisturbed, time-matched controls. The other time-matched controls with undisrupted sleep (i.e., ZT6, 10, and 14) were taken from the corresponding time-of-day matched samples described above.

### Tissue Collection.

Rats were decapitated by guillotine under normal room light (ZT2–10) or under dim red light (ZT14–22). Following decapitation, forebrains were resected (Fig. 1A), frozen in 2-methylbutane suspended in dry ice, and then stored at −80°C until homogenization for RNA extraction.

### RNA isolation.

Just before RNA isolation, forebrains were removed from − 80°C storage and placed on dry ice. Prior to use, a stainless-steel mortar and pestle were cleaned with RNase Zap (Thermo Fisher) and 70% ethanol. The mortar was then partially filled with liquid nitrogen before a forebrain was added, pulverized, and placed in a conical tube. Between each sample, the mortar and pestle were cleaned with 70% ethanol. A small aliquot of sample was removed for RNA isolation using Trizol Reagent (Invitrogen), according to the manufacturer’s instructions. Purified RNA was resuspended in water, and concentration and purity were measured with a Nanodrop spectrophotometer (Thermo Fisher). Samples were stored at −20°C until further processing was performed.

### Library preparation.

WTTS-seq libraries were prepared as described by Zhou et al.^27^. Briefly, total RNA (2.5 μg) was incubated at 70°C with 10X Fragmentation buffer (Invitrogen) for 3 min. The fragmentation reaction was halted by the addition of Stop Solution and incubation on ice for at least 2 min. Next, poly(A) + RNA was purified from the fragmented total RNA with Dynabeads Oligo (dT)25 (Invitrogen), according to the manufacturer’s directions, and used for first-strand cDNA synthesis in a 20 μL reaction mixture. First, 1.0 μL of barcode primer (100 μM) and 1.0 μL of a common SMART primer (100 μM) were annealed to the poly(A) + RNA template by heating to 65°C for 5 min and incubating on ice for at least 2 min. Next, 4.0 μL of 5X First-strand buffer (Invitrogen), 1.0 μL of SuperScript III reverse transcriptase (Invitrogen), 1.0 μL of 0.1 M dithiothreitol, 2.5 μL of 10 mM dNTP, and 1.0 μL of RNase OUT (Invitrogen) were added to the mixture. First-strand cDNA was synthesized by incubating the mixture at 40°C for 90 min in the presence of library-specific adaptors. Synthesis was terminated by heating the mixture at 70°C for 15 min. RNases I (100 U/μL; Invitrogen) and H (2 U/μL; Invitrogen) were subsequently added and incubated with the mixture at 37°C for 30 min to hydrolyze the remaining single-stranded RNA molecules and ensure that only single-stranded cDNA remained. RNase activities were terminated by heating the samples at 70°C for 20 min. Following purification with solid-phase reversible immobilization (SPRI) beads, second-strand cDNA was synthesized from first-strand cDNA by asymmetric PCR. In addition to the cDNA, the 50 μL PCR reaction contained 1.0 μL of Phusion Hi-Fidelity DNA polymerase, 10.0 μL of 5X HF buffer, 1.0 μL of 0.4 μM barcode primer, 1.0 μL of 0.8 μM common primer, 1.0 μL of 10 mM dNTP and nuclease-free water. The PCR reaction was carried out by heating at 95°C for 30 sec, followed by 20 cycles of 98°C for 10 s, 50°C for 30 s, and 72°C for 30 s, with a final elongation step at 72°C for 10 min. SPRI beads were used to purify and select 200–500 bp fragments from the final library. After quality control analyses, the size-selected library was sequenced with an Ion PGM Sequencer at the WSU Genomics Core Laboratory.

## Data analysis

### Raw read processing.

Raw data were obtained from 55 samples and stored in FASTQ format. We filtered raw reads with the FASTQ quality filter in the FASTX Toolkit (v0.0.13), allowing for a minimum score of ≥ 10 for ≥ 50% of bases (http://hannonlab.cshl.edu/fastx_toolkit/). We trimmed T nucleotides or T-rich sequences located at the 5’ ends of the reads using Perl scripts, as described previously^27^. Trimmed reads of at least 16 bp in length were kept for further analysis.

### Read mapping and poly(A) site clustering.

For each data set, the processed reads were aligned to the *Rattus norvegicus* genome (mRatBN7.2/rn7) using the torrent mapping program (TMAP, v3.4.1; http://github.com/iontorrent/tmap) with the unique best hits parameter (-a 0). Raw PASs supported by the uniquely mapped reads were extracted from SAM files and mergeded into a polyadenylation tag (PAT) file with a script previously used for WTTS-seq (freely available by contacting Dr. Zhihua Jiang, Washington State University). The PAT files were merged to determine the final PASs for all samples. PASs within 25 nucleotides of one another were grouped into one polyadenylation site cluster (PAC) using GetPolyaSiteCluster^77^. PACs were filtered taking into account the library size. For libraries that had less than 1.7M reads, PACs were required to have ≥ 1 set of 5 biological replicates had ≥ 3 samples with ≥ 3 reads. For libraries with more than 1.7M reads, at least 3 samples with ≥ 4 reads were required.

### Gene annotation and usage of poly(A) sites.

We annotated all the final PACs for PAS_ID, gene symbol, functional region, and other factors, as indicated, using Cuffcompare (v2.2.1)^78^, Perl scripts, and annotation file (GCF_000001895.5_Rnor_6.0_genomic.gtf; https://ftp.ncbi.nlm.nih.gov/). Clusters that mapped to mitochondrial genes were removed, then the number of PAS-covered reads was normalized^79^ to the total number of covered reads within each library and rescaled by a factor of 10^7^.

### Diurnal/ultradian PAS discovery.

Using normalized PAS read counts as input, rhythmic patterns were identified using the MetaCycle^32^ R package meta2d, which synthesizes the results of three cycle analysis algorithms (ARSER, JTK_Cycle, and Lomb-Scargle). The analysis was run 5 times with different replicates inserted into each of the appropriate ZT time slots^80^, and the median p-value, median BH.Q, average phase, amplitude, and relative amplitude were calculated. Only the highly corroborated PASs that were significant (*p* < 0.05) in all 5 trials were used for all analyses. Plots of read counts use normalized reads per 10^7^ and show the SEM of 5 biological replicates.

### Detailed mapping of APA sites.

The data supporting all figures depicting APA sites was from rat genome build BN7.2 and the UCSC (http://genome.ucsc.edu) and RDG (https://rgd.mcw.edu/rgdweb/homepage/) genome browsers^64,81^.)

### Gene ontology and pathway analysis.

Gene over-representation analysis was performed with the web-based tool WebGestalt^34^. Input gene symbol sets representing genes with cycling APA sites (*p* < 0.05 in 5 of 5 trials and > 1 PAS) or APA sites that were differentially expressed with sleep pressure (*p* < 0.01, log2FC > 0.5 and > 1 PAS), were compared to relevantly annotated rat genes using an output threshold of FDR ≤ 0.05. For phase-specific analysis, a sliding window of 5 h centered on each sample collection timepoint was used. For example, for phase ZT6, all PASs with average phase calculations that ranged from 3.5 to 8.5 were grouped.

### Differential expression analysis of sleep deprivation/recovery.

To evaluate the expression of PASs in sleep homeostasis experiments, PAS counts from rats recovering from 6 h SD were contrasted with time-matched controls (R0 vs ZT6, R2 vs ZT8, R4 vs ZT10, R8 vs ZT14). We removed high variation from the first principal component systematically, resulting in improved variance estimates for low read counts. Prcomp (in R) was used to perform principal component analysis (PCA) and to find eigenvectors by way of singular value decomposition. DESeq-2 with “Apeglm” Shrinkage^82^ and the Wald Test were used to generate test statistics in R software. The FDRtool was used to determine the Local FDR.

## Figures and Tables

**Table 1:** Diurnal APAs from genes with ≥ 2 APA sites (20 lowest median meta2d *p*-values). **PAS_ID** is a unique identifier for each PAS. Gene **Symbol, Chromosome** are listed, as well as **Strand** according to the convention of each chromosome. **Peak** is the mode or most common 3’ endpoint in the cluster. The number of **PASs per gene** is listed. **Total** refers to the sum of the reads for all samples. Meta2d calculations shown are: **median p** - median probability of cycling; **median BH.Q** - median Benjamini/Hochberg adjusted probability; **AVE phase** - average peak phase; **AVE Amp** - average amplitude (from peak to trough) of reads; and **AVE rAmp** - average relative amplitude adjusted by the mean read number. To look for functions or cell components that are particularly affected by APA site usage in a time-of-day dependent manner, we performed pathway and gene ontology (GO) over-representation analyses using the online tool WebGestalt^34^. The set of 1,173 gene symbols corresponding to diurnal PASs in genes with ≥ 2 APAs were input (Table 2 and Supplementary Table S3). Glutamatergic Synapse, Membrane Trafficking and Circadian Entrainment are among the enriched terms. In order to characterize genes with diurnal PASs and ≥ 2 total APAs, a corresponding set of 1,173 gene symbols were used as input into WebGestalt.

PAS_ID	Symbol	Chromosome	Strand	Peak	PASs/gene	Total	AVE p	AVE BH.Q	AVE phase	AVE Amp	AVE rAmp
497,844	Parvb	7	+	115,445,766	2	6,707	1.25E-10	1.69E-06	1.89	373	0.29
251,968	Dnajb1	19	−	24,522,731	2	4,379	9.60E-10	6.08E-06	15.54	194	0.24
424,776	Ccdc107	5	+	57,752,233	3	1,833	8.64E-09	3.01E-05	18.14	124	0.37
282,515	Rap1gds1	2	−	227,512,038	2	45	1.01E-08	3.31E-05	18.35	10	1.29
86,566	Hint1	10	+	38,993,254	3	44,628	1.65E-08	4.13E-05	11.56	1871	0.22
42,414	Dbp	1	+	96,180,614	2	5,424	2.52E-08	5.27E-05	11.88	408	0.40
496,957	Cacna1i	7	+	111,947,418	3	2,361	4.21E-08	7.48E-05	0.81	112	0.24
301,149	S100a10	2	+	179,229,660	2	863	5.20E-08	9.05E-05	21.50	88	0.59
249,570	Ndrg4	19	−	9,351,408	2	57,912	5.23E-08	9.30E-05	0.75	2119	0.19
141,758	Iars2	13	−	96,831,484	3	263	5.40E-08	9.42E-05	18.22	33	0.73
89,040	Phf23	10	+	54,722,784	2	2,014	6.26E-08	9.97E-05	13.48	119	0.32
455,360	Wdr35	6	+	31,831,183	5	154	6.30E-08	9.19E-05	18.05	23	0.83
279,711	Prpf38b	2	−	196,553,983	3	397	1.54E-07	1.91E-04	19.09	34	0.48
363,773	Cacna2d1	4	−	18,950,614	2	3,218	1.85E-07	2.00E-04	5.00	248	0.39
309,907	Cbs	20	−	9,708,260	4	3,085	2.07E-07	2.25E-04	21.42	165	0.30
214,595	Faf2	17	−	9,947,225	4	2,246	2.21E-07	2.44E-04	15.60	105	0.26
447,907	Syndig1l	6	−	104,323,418	2	253	2.56E-07	2.76E-04	8.92	36	0.77
470,887	Btbd11	7	−	18,035,151	3	3,401	2.97E-07	3.30E-04	23.59	184	0.29
449,908	Dicer1	6	−	123,627,529	3	296	4.49E-07	4.21E-04	17.00	30	0.58
549,543	Coq10b	9	+	56,573,671	2	334	5.45E-07	4.21E-04	16.60	32	0.54

**Table 2:** Gene ontology and pathway analysis of genes with diurnal APA site expression. The top 10 gene ontology terms and pathways identified by WebGestalt using the 1,173 genes with APAs that exhibited time-of-day oscillations and had 2 or more total APAs. **GO** and **pathway descriptions** are followed by the **size** (total number of genes that the term is comprised of), **overlap** (number of input genes matching the term), **expect** (number of input genes expected to match by chance), **ratio** (number of actual/expected matches), **pValue** (probability), **FDR** (false discovery rate; probability adjusted for multiple sampling), **database** (For GO analysis: BP, Biological process; CC, Cellular Component; MF, Molecular Function. For pathway analysis: KEGG, Kyoto Encyclopedia of Genes and Genomes; Panth, Panther; React, Reactome; Wiki, Wikipathway).

Gene ontology description	size	overlap	expect	ratio	pValue	FDR	database
neuron to neuron synapse	322	49	18.31	2.68	2.18E-10	2.09E-07	CC
postsynaptic specialization	327	47	18.59	2.53	3.49E-09	1.67E-06	CC
glutamatergic synapse	368	50	20.93	2.39	7.51E-09	2.39E-06	CC
glutamate receptor signaling pathway	78	19	4.44	4.28	4.87E-08	1.16E-05	BP
regulation of neuron projection development	422	52	24.00	2.17	1.01E-07	1.93E-05	BP
cell part morphogenesis	428	52	24.34	2.14	1.59E-07	2.54E-05	BP
positive regulation of cell projection organization	321	42	18.25	2.30	3.50E-07	4.79E-05	BP
synaptic membrane	396	47	22.52	2.09	1.25E-06	1.44E-04	CC
neuron spine	182	28	10.35	2.71	1.36E-06	1.44E-04	CC
positive regulation of cell component biogenesis	349	42	19.85	2.12	3.24E-06	3.10E-04	BP
Pathway description	size	overlap	expect	ratio	pValue	FDR	database
Axon guidance	216	31	11.17	2.77	2.20E-07	2.68E-04	React
Membrane Trafficking	457	50	23.64	2.12	3.73E-07	2.68E-04	React
Glutamatergic synapse	116	21	6.00	3.50	4.34E-07	2.68E-04	KEGG
Vesicle-mediated transport	483	51	24.99	2.04	8.44E-07	3.20E-04	React
Calcium Regulation in the Cardiac Cell	132	22	6.83	3.22	1.01E-06	3.20E-04	Wiki
Signaling by Receptor Tyrosine Kinases	304	37	15.73	2.35	1.04E-06	3.20E-04	React
Circadian entrainment	99	18	5.12	3.51	2.70E-06	7.15E-04	KEGG
MAPK family signaling cascades	218	28	11.28	2.48	7.76E-06	1.79E-03	React
Neuronal System	258	31	13.35	2.32	1.02E-05	2.09E-03	React
Signaling by VEGF	80	15	4.14	3.62	1.24E-05	2.27E-03	React

**Table 3:** GO and pathway terms associated with differentially expressed APA sites following sleep deprivation/recovery. GO and pathway analyses were performed on lists of genes with ≥ 2 APA sites that exhibited differential expression of at least 1 APA site following sleep deprivation/recovery compared to controls using the over-representation analysis function of the online web tool WebGestalt. GO or pathway description is followed by the **size** (total number of genes that the term is comprised of), **overlap** (number of input genes matching the term), **expect** (number of input genes expected to match by chance), **ratio** (number of actual/expected matches), **pValue** (probability), **FDR** (false discovery rate; probability adjusted for multiple sampling), **database** (For the GO analysis: BP, Biological process; CC, Cellular Component; MF, Molecular Function. For the pathway analysis: KEGG is Kyoto Encyclopedia of Genes and Genomes). **Comparison of APA-linked brain disorder susceptibility genes with WTTS-seq identified diurnal APAs and APAs differentially expressed with sleep pressure**. A recent survey by Cui et al.^31^ using APA transcriptome-wide association studies (TWAS) highlighted the importance of APA site usage in brain disorders. To establish the extent to which genes with APA-linked neurological phenotypes had diurnal or sleep related changes in rats, our list of diurnal genes with ≥ 2 APA sites was compared to those reported in Cui et al.^31^. There were 25 overlapping genes (representing 28 APAs in our data, since three genes had 2 diurnal APA sites). Another 19 genes with WTTS-seq-identified APA sites that cycle on a 12 h period were identified in the TWAS data set, as were nine genes (11 APA sites) that were differentially expressed with sleep pressure. Altogether, 54 APAs representing 46 genes were observed in common with genes having disease-associated APAs (Table 4).

ZT6 vs R0							
GO description	size	overlap	expect	ratio	pValue	FDR	database
synaptic membrane	396	5	0.37	13.40	1.88E-05	1.79E-02	CC
neuron to neuron synapse	322	4	0.30	13.18	1.69E-04	4.52E-02	CC
postsynaptic specialization	327	4	0.31	12.98	1.80E-04	4.52E-02	CC
localization within membrane	130	3	0.12	24.48	2.14E-04	4.52E-02	BP
glutamatergic synapse	368	4	0.35	11.53	2.83E-04	4.52E-02	CC
phosphatase binding	143	3	0.13	22.26	2.83E-04	4.52E-02	MF
ZT10 vs R4							
GO description	size	overlap	expect	ratio	pValue	FDR	database
synaptic membrane	396	31	8.09	3.83	1.22E-10	1.17E-07	CC
glutamatergic synapse	368	26	7.51	3.46	3.52E-08	1.68E-05	CC
neuron to neuron synapse	322	23	6.58	3.50	1.86E-07	5.85E-05	CC
postsynaptic specialization	327	23	6.68	3.44	2.45E-07	5.85E-05	CC
regulation of trans-synaptic signaling	375	24	7.66	3.13	7.43E-07	1.42E-04	BP
neuron spine	182	16	3.72	4.30	9.83E-07	1.57E-04	CC
glutamate receptor signaling pathway	78	10	1.59	6.28	3.94E-06	5.38E-04	BP
regulation of synapse structure or activity	201	15	4.10	3.65	1.60E-05	1.91E-03	BP
synaptic transmission, glutamatergic	83	9	1.69	5.31	4.73E-05	5.02E-03	BP
regulation of neuron projection development	422	22	8.62	2.55	5.46E-05	5.22E-03	BP
localization within membrane	130	11	2.65	4.14	7.10E-05	6.17E-03	BP
behavior	464	22	9.48	2.32	2.15E-04	1.71E-02	BP
positive regulation of nervous system development	447	21	9.13	2.30	3.39E-04	2.41E-02	BP
protein exit from endoplasmic reticulum	31	5	0.63	7.90	3.76E-04	2.41E-02	BP
synapse organization	296	16	6.04	2.65	3.86E-04	2.41E-02	BP
axon part	389	19	7.94	2.39	4.03E-04	2.41E-02	CC
presynapse	489	22	9.99	2.20	4.44E-04	2.50E-02	CC
cell part morphogenesis	428	20	8.74	2.29	5.02E-04	2.52E-02	BP
dendrite development	189	12	3.86	3.11	5.06E-04	2.52E-02	BP
regulation of G protein-coupled receptor signaling pathway	91	8	1.86	4.30	5.35E-04	2.52E-02	BP
ZT6 vs R0							
GO description	size	overlap	expect	ratio	pValue	FDR	database
cell leading edge	276	15	5.64	2.66	5.53E-04	2.52E-02	CC
endoplasmic reticulum to cytosol transport	21	4	0.43	9.33	7.73E-04	3.23E-02	BP
excitatory synapse	54	6	1.10	5.44	7.78E-04	3.23E-02	CC
endocytosis	418	19	8.54	2.23	9.62E-04	3.83E-02	BP
forebrain development	295	15	6.02	2.49	1.09E-03	4.18E-02	BP
receptor metabolic process	127	9	2.59	3.47	1.17E-03	4.31E-02	BP
ZT10 vs R4							
Pathway description	size	overlap	expect	ratio	pValue	FDR	database
mRNA surveillance pathway	95	11	1.81	6.07	1.83E-06	3.40E-03	KEGG

**Table 4:** APA-containing genes with diurnal or differentially expressed APAs detected in WTTS-seq that are that are also associated with human brain disorders. The number of **PASs per gene** is listed. **mPAS** is indicated if the PAS maps to a known, major PAS. refers to the sum of the reads for all samples. Meta2d calculations shown are: **median p** - median probability of cycling; **median BH.Q** - median Benjamini/Hochberg adjusted probability; **phase AVE** - average peak phase. Abbreviations for the brain disorders studied in Cui et al.^31^ are: amyotrophic lateral sclerosis (ALS), attention deficit hyperactivity disorder (ADHD), autism spectrum disorder (ASD), anxiety (ANX), bipolar disorder (BIP), depression (DEP), major depressive disorder (MDD), schizophrenia (SCZ), post-traumatic stress disorder (PTSD), Parkinson’s disease (PD) and Alzheimer’s disease (AD).

Cycle	PAS_ID	Symbol	Disorders Cui et al.	PASs/gene	major PAS	p AVE	BH.Q AVE	phase AVE
**24h**	550,479	Abi2	AD(1), DEP(1)	5		2.61E-03	1.14E-01	22
**24h**	126,847	Agfg2	AD(1)	2		1.04E-02	2.64E-01	21
**24h**	61,185	Arl3	ANX(1), BIP(2), SZC(6)	3		3.51E-02	5.26E-01	16
**24h**	234,171	Brd8	SCZ(2)	3		5.51E-03	1.81E-01	10
**24h**	188,939	Ccdc25	BIP(1)	6	mPAS	3.66E-02	5.37E-01	4
**24h**	82,922	Cdip1	SCZ(2)	3		9.97E-05	1.51E-02	17
**24h**	392,202	Chmp3	ANX(1)	4		9.61E-03	2.47E-01	7
**24h**	392,209	Chmp3	ANX(1)	4		3.11E-02	4.94E-01	18
**24h**	210,841	Ddhd2	SCZ(18)	2	mPAS	2.57E-03	1.11E-01	18
**24h**	330,991	Elp4	ANX(2), DEP(1)	2	mPAS	1.27E-03	7.33E-02	22
**24h**	331,015	Elp4	ANX(2), DEP(1)	2		2.49E-02	4.38E-01	1
**24h**	494,157	Emc2	ANX(11)	4	mPAS	5.60E-04	4.36E-02	16
**24h**	141,301	Enah	ANX(7), BIP(1)	4		1.51E-02	3.25E-01	21
**24h**	332,926	Frmd5	ANX(1), DEP(1)	6		1.15E-02	2.78E-01	2
**24h**	205,543	Gatad2a	SCZ(1)	3		9.60E-03	2.48E-01	19
**24h**	204,624	Grid1	ANX(1)	2		2.47E-04	2.66E-02	6
**24h**	121,855	Hip1r	SCZ(1), AD(1)	2		3.83E-02	5.54E-01	17
**24h**	61,332	Ina	SCZ(1)	3	mPAS	5.32E-03	1.76E-01	23
**24h**	351,590	Map1a	SCZ(1)	11		1.31E-05	4.05E-03	2
**24h**	95,493	Mapt	AD(1), ANX(2), ASD(1). DEP(2), MDD(2), PD(2), PTSD(2), SCZ(2)	9		1.13E-02	2.71E-01	1
**24h**	213,927	Ntrk2	ANX(1)	10	mPAS	2.02E-02	3.86E-01	20
**24h**	213,950	Ntrk2	ANX(1)	10		3.24E-02	5.05E-01	2
**24h**	55,226	Pnpla2	ADHD(1)	2		2.78E-03	1.18E-01	20
**24h**	514,594	Rbm6	DEP(1)	4	mPAS	2.06E-02	3.90E-01	18
**24h**	549,537	Sf3b1	SCZ(1)	4		8.47E-04	5.47E-02	8
**24h**	286,602	Ssbp2	PTSD(1)	5		1.03E-03	6.43E-02	17
**24h**	131,036	Svop	Anx(2)	4		8.34E-03	2.28E-01	15
**24h**	17,074	Wdr73	SCZ(2)	2		2.27E-02	4.09E-01	3
Cycle	PAS_ID	Symbol		PASs/gene	major PAS	p AVE	BH.Q AVE	phase AVE
**12h**	550,473	Abi2	AD(1), DEP(1)	5	mPAS	3.50E-02	6.33E-01	6
**12h**	61,185	Arl3	ANX(1), BIP(2), SZC(6)	3		1.84E-02	4.39E-01	5
**12h**	98,600	Cadm2	BIP(1)	16		3.40E-06	2.14E-03	5
**12h**	392,206	Chmp3	ANX(1)	4	mPAS	2.61E-02	5.18E-01	4
**12h**	287,927	Ercc8	SCZ(1)	2		8.29E-03	2.72E-01	6
**12h**	200,908	Fgfr1	SCZ(1)	5		2.82E-02	5.55E-01	8
**12h**	538,044	Hecw2	SCZ(1)	6		1.46E-04	2.16E-02	7
**12h**	61,328	Ina	SCZ(1)	3		1.75E-05	5.35E-03	11
**12h**	77,702	Lsm12	BIP(1)	2	mPAS	8.17E-03	2.58E-01	5
**12h**	529,393	Mon1a	DEP(1), MDD(1), PTSD(1)	2	mPAS	9.21E-04	7.38E-02	4
**12h**	525,326	Myo1e	SCZ(1)	2	mPAS	1.16E-02	3.17E-01	5
**12h**	90,245	Pitpna	AD(1)	8		2.01E-02	4.61E-01	2
**12h**	258,834	Pskh1	SCZ(1)	2		3.98E-03	1.76E-01	7
**12h**	145,571	Rab29	PD(1)	3	mPAS	3.33E-04	4.04E-02	6
**12h**	89,414	Rabep1	SCZ(13)	4		5.16E-06	2.72E-03	4
**12h**	22,342	Setd1a	PD(1)	2		2.67E-02	5.45E-01	3
**12h**	390,670	Snca	AD(1),PD(7),SCZ(3)	4		3.35E-03	1.57E-01	10
**12h**	18,304	Usp35	ANX(2)	2	mPAS	8.36E-04	6.91E-02	10
**12h**	218,787	Wac	BIP(1)	4		1.83E-05	5.61E-03	10
DE	PAS_ID	symbol		PASs/gene	major PAS	log2FC	pvalue	padj
**ZT10 vs R4**	467,701	Mark3	SCZ(1)	5		3.18	5.81E-09	6.94E-06
**ZT10 vs R4**	467,700	Mark3	SCZ(1)	5		3.00	7.13E-08	4.95E-05
**ZT10 vs R4**	191,750	Ndfip2	SCZ(1).BIP(1)	5		−1.15	2.55E-08	2.19E-05
**ZT10 vs R4**	218,787	Wac	BIP(1)	4		−1.51	1.63E-04	1.30E-02
**ZT10 vs R4**	549,537	Sf3b1	SCZ(1)	4		−1.30	6.47E-04	3.15E-02
**ZT10 vs R4**	35,839	Mtrf1l	SCZ(1)	4		−1.60	9.64E-04	4.08E-02
**ZT10 vs R4**	205,547	Gatad2a		3	mPAS	1.03	9.24E-05	9.16E-03
**ZT10 vs R4**	205,543	Gatad2a	SCZ(1)	3		1.42	2.29E-03	7.24E-02
**ZT10 vs R4**	261,774	Spg7	SCZ(2)	3	mPAS	2.17	2.75E-04	1.89E-02
**ZT10 vs R4**	519,450	Snx19	ADHD(1), BIP(1), SCZ(3)	3		−1.18	3.65E-04	2.20E-02
**ZT10 vs R4**	78,129	Plekhm1	ANX(1), DEP(1), PD(1), PTSD(1), SCZ(1)	3	mPAS	1.96	2.59E-03	7.74E-02

## Data Availability

The PAS sequence data discussed here have been deposited in NCBI’s Gene Expression Omnibus and are accessible through GEO Series accession number GSE250324 (https://www.ncbi.nlm.nih.gov/geo/query/acc.cgi?acc=GSE250324).
